# Thromboprophylaxis in Intermediate and Major Plastic Surgery Procedures: A Narrative Review and Proposal of the TROMBO-PLAST Integrated Decision-Making Model

**DOI:** 10.7759/cureus.110817

**Published:** 2026-06-14

**Authors:** Luiz Augusto Sousa Oliveira, Ligia Helena Mendes, Nélio Nunes Cabette Filho, Victor da Costa Sacksida Valladão

**Affiliations:** 1 General Surgery, Fundação Hospitalar Santa Terezinha (FHSTE), Erechim, BRA; 2 Plastic Surgery, Hospital Niterói D'Or, Serviço de Pós-Graduação em Cirurgia Plástica Prof. Ronaldo Pontes, Niterói, BRA

**Keywords:** body contouring, caprini score, low-molecular-weight heparin, plastic surgery, pulmonary embolism, venous thromboembolism

## Abstract

Venous thromboembolism (VTE) is a leading cause of preventable perioperative morbidity and mortality in plastic surgery. However, its prevention demonstrates considerable variation in clinical practice, ranging from under-prophylaxis driven by concerns about hematoma formation to routine over-prophylaxis based solely on numerical risk scores. This narrative review critically examines the available tools for thrombotic and bleeding risk stratification in intermediate and major plastic surgery procedures and proposes an integrated decision-making model tailored to the specialty. A literature search was conducted in PubMed/MEDLINE, Cochrane Library, Embase, and SciELO, with priority given to publications from 2020 to 2026, complemented by selected landmark references. The Caprini score remains the most extensively validated risk stratification tool; however, it is insufficient when used in isolation. Integration with bleeding risk assessment, recognition of specialty-specific risk factors (including large-volume liposuction, abdominoplasty, gluteal fat grafting, post-bariatric body contouring, microsurgical flaps, and combined procedures), universal mechanical prophylaxis measures, appropriate timing of low-molecular-weight heparin administration, and dynamic postoperative reassessment can facilitate safer clinical decision-making. Thromboprophylaxis in plastic surgery requires the integration of thrombotic risk, bleeding risk, procedural context, and postoperative reassessment. The TROMBO-PLAST model is proposed as a practical framework to support this clinical reasoning while preserving individualized clinical judgment.

## Introduction and background

Venous thromboembolism (VTE), encompassing deep vein thrombosis (DVT) and pulmonary embolism (PE), remains one of the leading causes of preventable perioperative morbidity and mortality [1]. In elective aesthetic plastic surgery, PE ranks among the principal causes of postoperative death [2,3]. Despite the wide availability of evidence-based guidelines [1], thromboprophylactic decision-making in clinical practice continues to show substantial variation, oscillating between two equally problematic extremes: under-prophylaxis, driven by concerns about hematoma formation, and the automatic application of standardized regimens based solely on numerical scores, without consideration of surgical and individual patient factors [4].

Intermediate and major plastic surgery procedures present particular challenges that distinguish their prophylactic management from that of general surgery [4,5]. These include prolonged operative times, patient positioning that may compromise venous return, infusion of large volumes of tumescent solution, a predominantly female population with frequent use of oral contraceptives or hormone replacement therapy, and extensive tissue undermining, all factors that alter the balance between thrombotic and bleeding risk.

In the Brazilian context, characterized by heterogeneity among hospital services, private clinics, outpatient procedures, and the availability of institutional protocols [6], practical and adaptable models may help support a minimum standardization of perioperative safety.

Beyond these procedure-specific considerations, three conceptual gaps further justify an integrated approach. First, the Caprini score estimates thrombotic risk but does not quantify bleeding risk, a critical limitation in a specialty where hematoma and reoperation have substantial implications for both functional and aesthetic outcomes [4,5]. Second, the balance between thrombotic and bleeding risk is not static. Postoperative events such as hematoma, reintervention, infection, and immobilization may alter this balance, necessitating ongoing reassessment rather than a single preoperative decision [7-10]. Third, combined procedures and post-bariatric body contouring are associated with risks up to fivefold higher than those of isolated procedures, requiring a distinct decision-making framework [11].

This article aims to critically review the risk-stratification tools currently available for plastic surgery; systematize an integrated decision-making flowchart encompassing thrombotic risk, bleeding risk, and dynamic reassessment; and propose the TROMBO-PLAST model, which organizes thromboprophylaxis in the specialty across six interdependent dimensions.

A narrative review methodology was adopted because of the heterogeneity of study designs, populations, procedures, and outcomes evaluated. PubMed/MEDLINE, Cochrane Library, Embase, and SciELO were searched, with the latest search conducted in May 2026. Publications from 2020 to 2026 were prioritized, with complementary inclusion of seminal earlier studies. Search terms included “venous thromboembolism”, “thromboprophylaxis”, “plastic surgery”, “abdominoplasty”, “liposuction”, “gluteal fat grafting”, “body contouring”, and “Caprini”, used individually and in Boolean combinations. Study selection prioritized methodological quality, recency, and direct applicability to intermediate and major plastic surgery. Principal society guidelines and task-force recommendations relevant to surgical thromboprophylaxis, notably those of the American Society of Hematology and the American Society of Plastic Surgeons VTE Task Force, were included, and no major specialty-society recommendations were intentionally excluded [1,3]. Where plastic surgery-specific data were unavailable, evidence was extrapolated from general surgery, abdominopelvic oncologic surgery, and orthopedics; such instances are explicitly identified throughout the text.

Eligibility followed prespecified criteria. We included randomized controlled trials, prospective and retrospective cohort studies, systematic reviews, meta-analyses, clinical practice guidelines, and pivotal case series addressing thrombotic risk, bleeding risk, or thromboprophylaxis in intermediate and major plastic surgery. When specialty-specific data were unavailable, landmark studies from general, oncologic, and orthopedic surgery were included as extrapolated evidence. We excluded isolated case reports, opinion pieces lacking primary data, and studies confined to minor or office-based procedures without relevance to the target population. A representative search strategy, adapted syntactically to each database, combined the following terms: (“venous thromboembolism” OR “deep vein thrombosis” OR “pulmonary embolism” OR thromboprophylaxis) AND (“plastic surgery” OR abdominoplasty OR liposuction OR “gluteal fat grafting” OR “body contouring”) AND (Caprini OR “risk assessment” OR “bleeding risk” OR enoxaparin OR “mechanical prophylaxis”). Titles and abstracts were screened for thematic relevance by the authors. Potentially eligible articles underwent full-text review, and the reference lists of retrieved studies were hand-searched for additional sources. Seminal pre-2020 references were retained when they established foundational concepts or instruments (e.g., the original Caprini risk-assessment model and pivotal extended-prophylaxis trials) or when no more recent equivalent evidence was available.

## Review

Thrombotic risk stratification

The Caprini score (2005, 2010, and 2013 versions) is the most extensively validated risk-stratification tool in surgical populations [12-14]. In plastic surgery, Pannucci et al. further established its validity in a series comprising more than 8,000 patients, demonstrating a consistent correlation between increasing score ranges and the incidence of symptomatic VTE [15-17]. A clinically meaningful inflection point appears at a Caprini score of ≥7, where the 60-day postoperative incidence of VTE may exceed 6% in the absence of chemoprophylaxis, with postoperative enoxaparin reducing this risk by approximately 60% [9,15]. Importantly, this benefit is not uniform across all risk categories. In an individual-patient meta-analysis by Pannucci et al., a clear reduction in symptomatic VTE with chemoprophylaxis was observed primarily in patients with a Caprini score of 7-8 or higher [9]. In contrast, among patients in the moderate-risk range (Caprini 3-6), the absolute benefit of routine anticoagulation was smaller and less definitive and therefore must be weighed against the potential risk of bleeding [9]. This gradient forms the rationale for the deliberately conservative chemoprophylaxis thresholds, in which pharmacological prophylaxis becomes routine only in the high-risk category and above.

The score, however, should be interpreted with caution. A recent simulation analysis showed that most absolute VTE events occur in patients with a Caprini score of ≤6, reflecting the greater proportion of patients within this range in aesthetic surgery populations [10]. In addition, concerns have been raised regarding the discriminatory performance of the score in elective aesthetic surgery [18]. Nevertheless, the position advocated in this review is that the Caprini score remains the best-validated risk-stratification tool available for plastic surgery. Its principal limitation lies not in the score itself, but in its isolated and decontextualized application. Table 1 presents a plastic surgery-specific risk stratification framework and the corresponding management recommendations for each category, with a deliberately cautious approach in intermediate-risk patients.

**Table 1:** Caprini-based thrombotic risk stratification calibrated for intermediate and major plastic surgery procedures and suggested management *Approximate 60-day VTE incidences without prophylaxis are adapted from Pannucci et al.'s plastic and reconstructive surgery validation series for the moderate-to-extreme categories (Caprini ≥ 3) [15-17]; values for the very-low and low categories (Caprini 0-2) are extrapolated from general surgical populations and reflect expert consensus. The strongest evidence for a net benefit of pharmacological prophylaxis derives from high-risk patients (Caprini ≥ 7-8) [9,15]; the suggested management for the intermediate categories (Caprini 3-6) is therefore a deliberately conservative expert-consensus recommendation. IPC: intermittent pneumatic compression, GCS: graduated compression stockings, LMWH: low-molecular-weight heparin, OC: oral contraceptive, HRT: hormone replacement therapy. Source: Adapted from the Caprini risk assessment model [12-14].

Caprini (points)	Category	Approximate 60-day VTE incidence without prophylaxis*	Suggested management	Evidence basis
0-1	Very low	<0.5%	Early ambulation; intraoperative mechanical prophylaxis if immobilization > 1 h or at-risk positioning	Extrapolated from general-surgery data; expert consensus
2	Low	≈0.5% to 1.5%	Universal mechanical prophylaxis (IPC/GCS) intraoperatively until full ambulation	Extrapolated from general-surgery data; expert consensus
3-4	Moderate	≈1.5% to 3%	Universal mechanical prophylaxis; consider 7-day LMWH only if additional plastic surgery-specific factors are present (prolonged time, active OC/HRT, large-volume liposuction, associated abdominoplasty, or reduced mobility)	Plastic surgery validation series [15-17]; cautious expert-consensus threshold
5-6	High	≈3% to 6%	Mechanical + LMWH (enoxaparin 40 mg/day SC) for 7-10 days, started 12-24 h postoperatively with confirmed hemostasis	Plastic surgery validation series [15-17]
≥7	Very high/extreme	≥6%	Mechanical + prophylactic-dose LMWH + consider EXTENDED prophylaxis (14-28 days)	Direct plastic surgery evidence [9,15]; strongest support for chemoprophylaxis

Bleeding risk

Unlike thrombotic risk, perioperative bleeding risk in plastic surgery lacks a universally validated risk-assessment tool. The IMPROVE-Bleed model [19], originally developed for hospitalized medical patients, provides a useful conceptual framework. Variables such as moderate-to-severe renal failure, thrombocytopenia (<100,000/mm³), age ≥85 years, male sex, and active cancer contribute to bleeding risk within the model. However, its application in plastic surgery requires integration with specialty-specific factors (Table 2).

**Table 2:** Bleeding risk factors relevant to intermediate and major plastic surgery procedures Source: Based on the IMPROVE-Bleed model [19] and plastic surgery bleeding data [20].

Domain	Factors
Patient	Advanced age; poorly controlled hypertension; known coagulopathy; moderate/severe renal failure; prior use of antiplatelet or anticoagulant agents; history of hematoma in previous surgery
Procedure	Extensive undermining; wide aponeurotic plication; large-volume liposuction; extensive flaps; combined procedures; microsurgical reconstructions
Intraoperative	Hypothermia (core temperature < 36 °C); diffuse bleeding; prolonged operative time; technical difficulty with hemostasis
Postoperative	Uncontrolled postoperative hypertension; intense vomiting and coughing; agitation; increased serosanguineous drainage; expanding early hematoma

A recent systematic review of abdominoplasty reported a hematoma rate of 4.7% in anticoagulated patients compared with 0.6% in non-anticoagulated patients, a statistically significant difference [20]. This magnitude of effect illustrates the clinical challenge and reinforces the need for explicit bleeding-risk assessment. Conversely, a prospective series of lipoabdominoplasty patients managed with a combined protocol (intraoperative mechanical prophylaxis plus delayed initiation of enoxaparin) reported hematoma and PE rates of 1.8% and 0.6%, respectively, suggesting that protocol design, rather than the mere use of anticoagulation, is a major determinant of outcomes [21]. Taken together, these studies highlight an important lesson for the specialty: anticoagulation alone is not synonymous with safety.

Bleeding and thrombosis are not antagonistic phenomena. Hypothermia simultaneously impairs hemostasis by prolonging coagulation times and compromises microcirculation by promoting venous stasis [7,22]. This dual effect justifies the inclusion of normothermia among the adjunctive measures of the proposed model. Table 3 summarizes representative clinical scenarios, their impact on the thrombotic and bleeding axes, and the corresponding management recommendations.

**Table 3:** Integrated decision (thrombotic risk × bleeding risk): representative scenarios LMWH: low-molecular-weight heparin, IPC: intermittent pneumatic compression, OC: oral contraceptive, HRT: hormone replacement therapy. Source: Authors' synthesis integrating thrombotic [9] and bleeding [19] risk-assessment frameworks.

Scenario	Thrombotic risk	Bleeding risk	Suggested practical management
Caprini ≥ 7 with safe hemostasis	High	Variable	LMWH 12-24 h postoperatively + consider extended prophylaxis
Extensive undermining, borderline hemostasis	Moderate	High	Start LMWH only after secure hemostasis (24-48 h); maintain continuous IPC
Persistent intraoperative hypothermia	Increased stasis/poor perfusion	Increased coagulopathy	Normothermia as a mandatory measure; postpone LMWH until thermal stability
Reoperation or expanding hematoma	High due to inflammation/immobility	High	Suspend LMWH; reassess every 24 h; maintain mechanical prophylaxis
Active OC/HRT not suspended	High	Not necessarily high	Treat as increased thrombotic risk; discontinue if feasible before the next surgical step
Post-bariatric/body contouring	High	Variable	Consider extended prophylaxis up to 28 days
Active cancer/microsurgical reconstruction	High	Variable	Consider extension up to 4 weeks

Prophylactic strategies

Intermittent pneumatic compression (IPC) and graduated compression stockings (GCS) reduce the incidence of VTE by approximately 60% without increasing bleeding complications, based on evidence derived largely from general and mixed surgical populations [23]. These measures should be initiated before anesthetic induction and maintained continuously until full ambulation is achieved.

Subcutaneous enoxaparin, administered at a dose of 40 mg once daily (or 30 mg twice daily in very high-risk patients), is the low-molecular-weight heparin (LMWH) of choice [15,21]. The timing of initiation is critical. Preoperative administration is associated with an increased risk of hematoma in abdominoplasty and extensive flap procedures, whereas initiation 12-24 hours postoperatively, after confirmation of adequate hemostasis, has a more favorable safety profile. This approach has been associated with a reduction in symptomatic VTE without a significant increase in reoperations for hematoma [15,21]. Treatment should be maintained for at least seven days in patients at moderate-to-high risk and extended to 14-28 days in post-bariatric body-contouring procedures and selected oncologic cases [24,25]. Additional dose adjustments should be considered in patients with renal impairment (creatinine clearance < 30 mL/min) and severe obesity (BMI > 40 kg/m²), in whom altered pharmacokinetics may necessitate dose modification under specialist supervision [26]. Table 4 summarizes situations in which LMWH should be temporarily withheld.

**Table 4:** Situations in which LMWH chemoprophylaxis should be temporarily withheld LMWH: low-molecular-weight heparin. Source: Based on enoxaparin dosing and perioperative timing evidence [15,26].

Item	Criterion
Hemostasis	Insecure intraoperative hemostasis or significant serosanguineous drainage
Hematoma	Clinically or ultrasound-evident expanding hematoma
Reoperation	Anticipated need for surgical reapproach within the next 24 h
Blood pressure	Uncontrolled postoperative hypertension (systolic BP > 160 mmHg)
Hematology	Relevant thrombocytopenia (<80,000/mm³) or clinical/laboratory coagulopathy
Thermal	Persistent hypothermia with diffuse bleeding
Anesthesia	Recent neuraxial blockade: respect the window per agent class and dose

Extended post-discharge prophylaxis deserves particular emphasis. The ENOXACAN II trial, conducted on patients undergoing abdominopelvic cancer surgery, demonstrated a 60% reduction in VTE, with extension of prophylaxis to four weeks [24]. This principle has been successfully extrapolated to post-bariatric body-contouring procedures, in which thrombotic risk remains elevated for several weeks after discharge [25,27].

Direct oral anticoagulants (DOACs) may represent a potential alternative in selected scenarios requiring extended post-discharge prophylaxis, particularly when bleeding risk is low and adherence to LMWH is expected to be challenging. However, evidence specific to plastic surgery remains limited, with most available data extrapolated from orthopedic surgery [28]. Therefore, routine incorporation of DOACs into plastic surgery thromboprophylaxis protocols cannot currently be recommended until specialty-specific studies become available.

Procedure-specific recommendations

Although individualized clinical judgment is irreplaceable, stratification by procedure type helps calibrate risk expectations and guide prophylactic decision-making. Table 5 summarizes baseline risk and suggested management strategies for the most common plastic surgery procedures, including combined procedures, a scenario in which the cumulative risk is not simply additive. Indeed, VTE-related mortality following combined procedures may be up to threefold higher in some series [11,27].

**Table 5:** Risk particularities and suggested management by procedure type in plastic surgery BBL: Brazilian butt lift. Source: Based on combined-procedure [11,27] and gluteal fat grafting [29,30] data.

Procedure	Baseline risk	Key particularities	Suggested management
Isolated liposuction (<5 L)	Low	Tumescent; usually <2 h	Intraoperative mechanical + early ambulation
Large-volume liposuction (≥5 L)	Moderate	Hypothermia, fluid shifts, prolonged operative time	Mechanical + 7-day LMWH if Caprini is ≥5
Isolated abdominoplasty	Moderate-high	Flexed positioning; plication; wide undermining	Mechanical + LMWH 7-10 days, started 12-24 h postoperatively
Lipoabdominoplasty	High	Combines factors of both	Mechanical + LMWH ≥ 7-10 days; reassess for extension
Post-bariatric body contouring	Very high	Multiple sites, high BMI, >6 h operative time	Mechanical + extended LMWH up to 28 days
Gluteal fat grafting (BBL)	Specific (high)	Fat embolism; prone positioning; deep cannulas	Intraoperative mechanical; subcutaneous-only injection with cannula ≥ 4 mm; LMWH per Caprini
Breast reconstruction with microsurgical flap	High	>6 h operative time; associated oncologic resection	Mechanical + LMWH ≥ 10 days; extended to 28 days if active cancer
Facelift in at-risk patient/prolonged rhytidectomy	Based on Caprini	Semi-seated position; >4 h; hormonal profile	Universal mechanical; LMWH only if Caprini is ≥5
Combined procedures	High	Prolonged time, multiple sites, greater immobility	Universal mechanical; LMWH if Caprini is ≥5 or additional factors; reassess for extension

**Figure 1:**
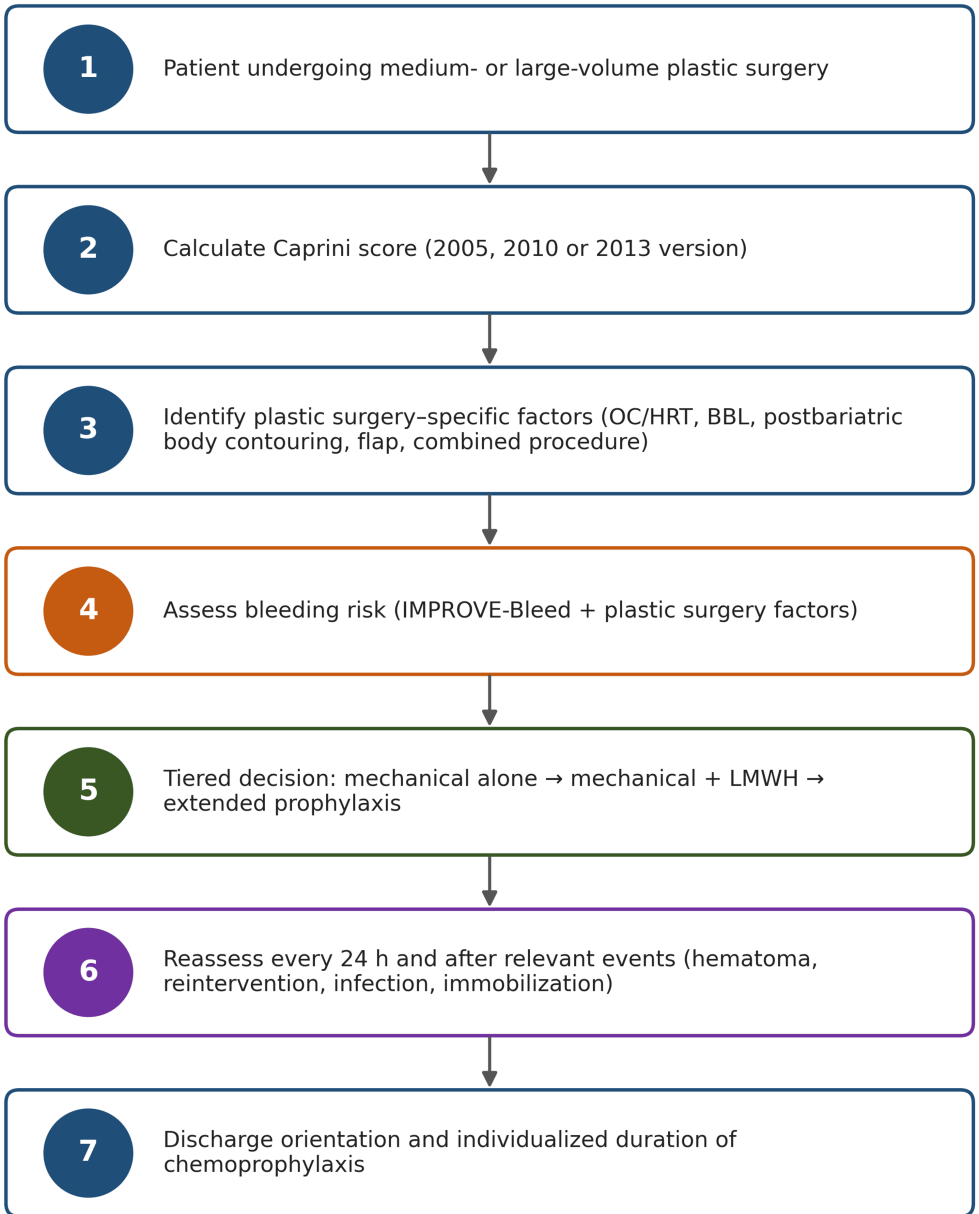
Integrated decision-making flowchart for thromboprophylaxis in plastic surgery Integrated decision-making flowchart for thromboprophylaxis in intermediate and major plastic surgery procedures. Each block represents a sequential decision step, with mandatory 24-hour reassessment at step 6. IPC: intermittent pneumatic compression, GCS: graduated compression stockings, LMWH: low-molecular-weight heparin, OC: oral contraceptive, HRT: hormone replacement therapy. Note: This image was created by the authors integrating the Caprini [9] and IMPROVE-Bleed [19] frameworks.

Figure 1 summarizes the integrated decision-making process, from initial risk stratification through dynamic reassessment, in a practical bedside-ready flowchart.

Gluteal fat grafting: VTE and fat embolism are distinct phenomena

Gluteal fat grafting (Brazilian butt lift, BBL) occupies a unique position in this discussion. Pulmonary fat embolism resulting from inadvertent intramuscular or subfascial fat injection represents the procedure’s characteristic fatal complication, with historically estimated mortality rates ranging from 1:3,000 to 1:6,000. Importantly, a consistent decline in mortality has been observed following the widespread adoption of subcutaneous-only fat injection techniques [29,30].

It is essential to distinguish conceptually between pulmonary fat embolism and classic VTE. Fat embolism associated with gluteal fat grafting is not a form of classic VTE, and anticoagulant prophylaxis does not prevent intravascular fat injection. Prevention depends primarily on adherence to safe surgical techniques, including exclusive use of the subcutaneous plane, cannulas ≥ 4 mm in diameter, continuous awareness of cannula position, and, ideally, intraoperative ultrasound guidance [30]. The conventional thrombotic risk associated with BBL coexists with the risk of fat embolism and therefore requires independent thromboprophylactic assessment based on the Caprini score and other procedure-specific risk factors. One risk does not substitute for the other, and each must be addressed through its respective preventive strategy.

Special situations

Combined estrogen-progestin oral contraceptives increase the risk of VTE by approximately two- to fourfold, while hormone replacement therapy is associated with a two- to threefold increase in risk [31]. Discontinuation is ideally recommended four weeks before elective intermediate or major surgery. However, the decision to suspend therapy must take into account competing risks, including unplanned pregnancy and worsening menopausal symptoms, and should be made through informed discussion with the patient.

A personal history of VTE contributes 3 points to the Caprini score. Hereditary thrombophilias, including factor V Leiden, the prothrombin G20210A mutation, and deficiencies of protein C, protein S, or antithrombin, as well as acquired thrombophilias such as antiphospholipid syndrome, warrant preoperative hematologic evaluation and frequently justify extended thromboprophylaxis. Outpatient procedures lasting more than three hours in patients with cumulative risk factors may also require chemoprophylaxis based on the same decision-making framework, with particular attention to patient education, home monitoring, and timely access to reassessment.

The TROMBO-PLAST model: an integrated proposal

**Figure 2:**
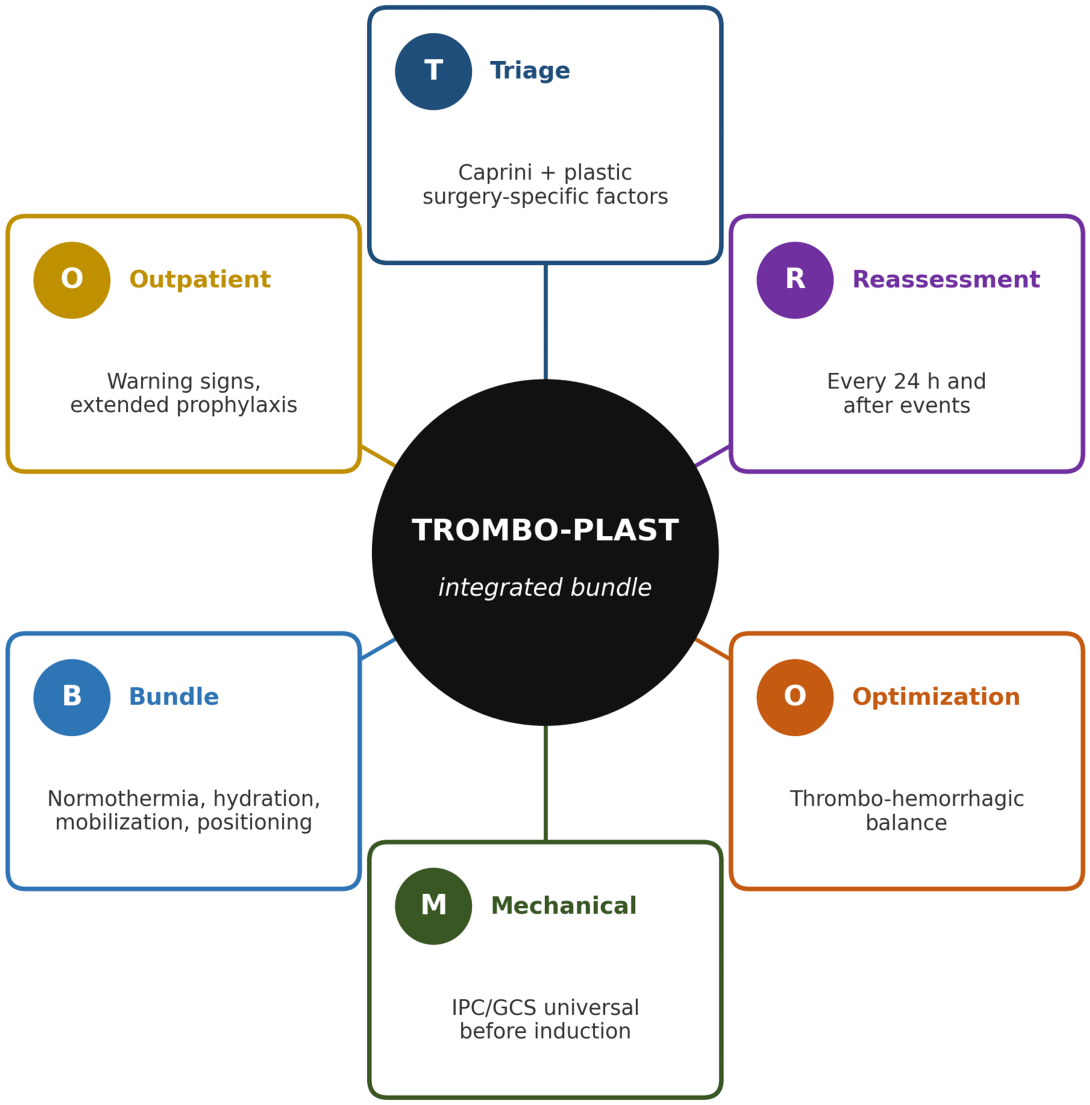
TROMBO-PLAST model: proposal of a six-dimension interdependent decision bundle for personalized thromboprophylaxis in plastic surgery T: triage, R: reassessment, O: optimization, M: mechanical prophylaxis, B: bundle of adjuvant measures, O: outpatient orientation and extended prophylaxis, IPC: intermittent pneumatic compression, GCS: graduated compression stockings. Note: This image was created by the authors, summarizing the model proposed in this review and integrating the frameworks discussed [7,9,19,21,23].

The evidence reviewed, together with the identified knowledge gaps, supports an approach that moves beyond the binary question of “prophylaxis versus no prophylaxis.” We therefore propose the TROMBO-PLAST model, a six-dimension, interdependent decision framework for plastic surgery practice, illustrated in Figure 2, and described in narrative form below. Unlike the preceding sections, which synthesize published evidence, the TROMBO-PLAST model represents an expert-opinion proposal that seeks to organize and operationalize the available evidence. It is presented as a practical decision-support framework rather than a validated instrument. Prospective evaluation will be necessary before any claims can be made regarding its effectiveness or superiority over current practice.

The first dimension, triage (T), consists of preoperative risk stratification using the Caprini score, complemented by specialty-specific factors, including active use of oral contraceptives or hormone replacement therapy, gluteal fat grafting, microsurgical flap reconstruction, post-bariatric body contouring, and a personal history of VTE. The second dimension, reassessment (R), requires reassessment every 24 hours during hospitalization and following clinically relevant events such as hematoma, reintervention, infection, or prolonged immobilization. The third dimension, optimization (O) of the thrombotic-bleeding balance, incorporates the IMPROVE-Bleed framework together with bleeding risk factors specific to plastic surgery. The fourth dimension, mechanical prophylaxis (M), is a universal measure consisting of IPC and/or GCS, initiated before anesthetic induction and maintained continuously until full ambulation in intermediate and major procedures. The fifth dimension, the bundle of adjunctive perioperative measures (B), includes maintenance of normothermia (core temperature ≥ 36 °C), adequate hydration, early mobilization within 24 hours, positioning that avoids venous compression, and discontinuation of oral contraceptives or hormone replacement therapy before surgery when feasible. The sixth dimension, outpatient orientation and extended prophylaxis (O), encompasses patient education, recognition of warning signs of DVT and PE, and individualized determination of chemoprophylaxis duration, typically 14 days in high-risk patients and up to 28 days in post-bariatric body-contouring procedures or cases associated with malignancy, with possible transition to a DOAC in selected cases.

The integration of these six dimensions seeks to address common gaps in clinical practice, particularly the lack of dynamic reassessment and the underuse of adjunctive preventive measures. Perioperative normothermia, considered in isolation, simultaneously reduces thrombotic risk by minimizing microcirculatory stasis and bleeding risk by preserving hemostatic function [7,22]. It therefore provides a paradigmatic example of the integrated clinical reasoning that the model seeks to operationalize.

Quality assurance and post-discharge orientation

Institutionalizing thromboprophylaxis requires the use of measurable and traceable quality indicators. Five essential indicators are proposed: the proportion of patients with a documented Caprini score, the proportion with a documented bleeding risk assessment, the proportion with IPC applied before anesthetic induction, the rate of hematoma requiring reoperation, and the rate of symptomatic VTE at 30 and 60 days. These indicators are summarized in Table 6, together with key elements of post-discharge patient education, including recognition of the signs and symptoms of DVT (pain, asymmetric edema, and calf swelling) and PE (sudden dyspnea, chest pain, unexplained tachycardia, and syncope), adherence to the prescribed prophylactic regimen, and prompt medical evaluation in the event of symptom onset.

**Table 6:** Quality assurance: post-discharge orientation and institutional indicators DVT: deep vein thrombosis, LMWH: low-molecular-weight heparin, VTE: venous thromboembolism. Source: Indicators proposed by the authors, informed by current VTE prevention guidelines [1,3].

Domain	Content
Post-discharge orientation (patient)	Early and frequent ambulation; adequate hydration; avoid prolonged sitting/lying; guidance for prolonged travel; recognize DVT signs (pain, asymmetric edema, calf engorgement) and PE signs (sudden dyspnea, chest pain, unexplained tachycardia, syncope); adherence to prescribed LMWH/anticoagulant; prompt return upon symptoms
Suggested institutional indicators	(i) percentage of patients with documented Caprini score in the chart; (ii) percentage with documented bleeding risk assessment; (iii) percentage with IPC applied before anesthetic induction; (iv) rate of hematoma requiring reoperation; (v) symptomatic VTE rate at 30 and 60 days

Limitations

This review has limitations inherent to its narrative design. No prospectively registered protocol was used, the number of records retrieved and screened was not formally reported using a PRISMA-style selection process, and no formal instrument-based risk-of-bias assessment of individual studies (e.g., RoB 2, ROBINS-I, or AMSTAR 2) was performed. Consequently, a quantitative synthesis was not undertaken. The findings should therefore be interpreted as a structured and transparent expert synthesis rather than as a systematic review. In addition, some recommendations applied to plastic surgery are derived from studies conducted in general, oncologic, orthopedic, or reconstructive surgical populations, and should therefore be interpreted with appropriate caution when extrapolated to plastic surgery practice. The TROMBO-PLAST model represents a conceptual and practical framework that has not yet undergone prospective validation. Its institutional implementation should be accompanied by systematic outcome monitoring to enable ongoing evaluation and iterative refinement.

## Conclusions

Thromboprophylaxis in intermediate and major plastic surgery procedures requires integration between thrombotic stratification, bleeding assessment, surgical context, and postoperative reassessment. Current evidence does not support either defensive under-prophylaxis or indiscriminate automatic prophylaxis. The flowchart and the TROMBO-PLAST model proposed here do not replace clinical judgment; they organize it. Their incorporation into institutional routine, combined with systematic chart documentation and event auditing, offers a concrete path to reduce variability among providers, prevent VTE, and simultaneously reduce avoidable hematomas. Transforming thromboprophylaxis into a quality-of-care process is a collective task of the specialty.
